# Supplementary material for: Longitudinal associations of an exposome score with serum metabolites from childhood to adolescence
Source: Commun Biol. 2024 Jul 22;7:890. doi: 10.1038/s42003-024-06146-0 (PMC11263428; doi:10.1038/s42003-024-06146-0)
Supplement: Supplementary file 2 — Supplementary Information [file 42003_2024_6146_MOESM2_ESM.pdf]

Supplementary Figure S1. Comparison of the significant associations of exposome models using different diet scores in the formulation of the exposome score with serum metabolites measured by LC-MS

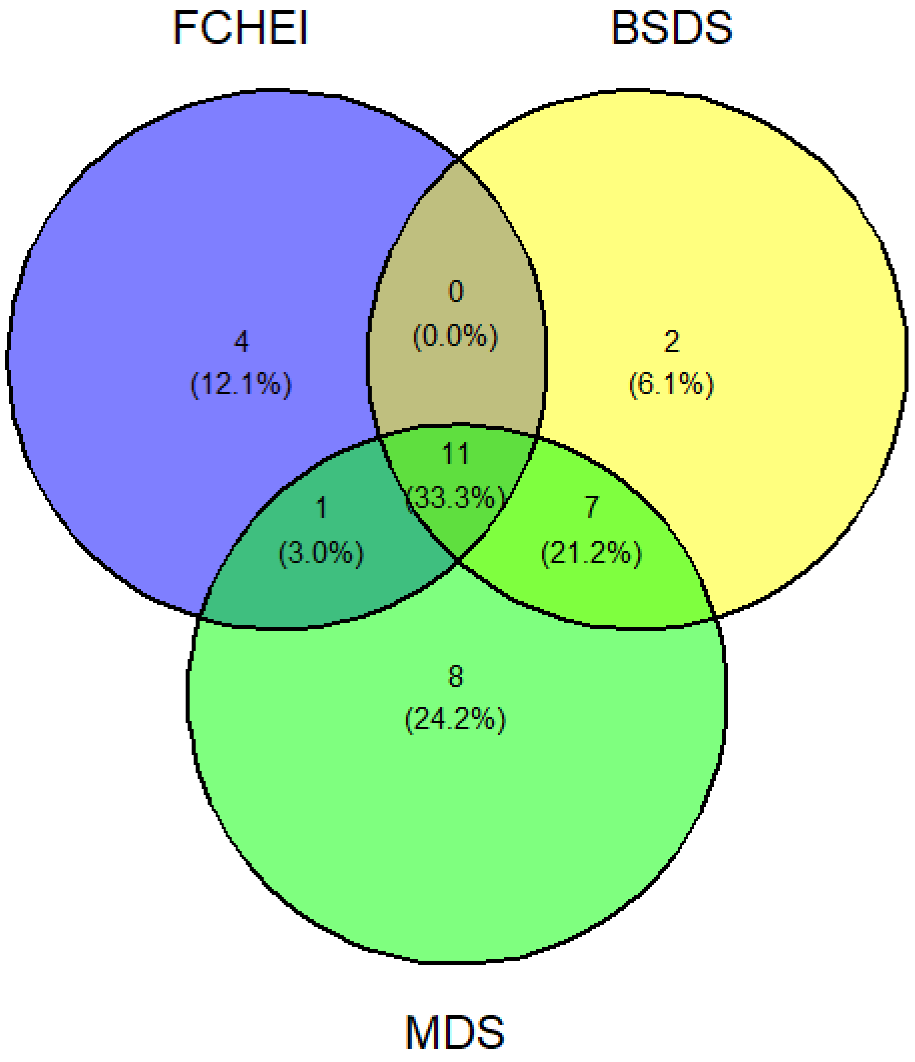

The exposome score (ranging between 5 and 20) was computed by summing up quartile scores for diet quality (measured by Finnish children healthy eating index), activity (combination of total physical activity and sedentary time), pollution exposure (combination of nitrogen oxide, nitrogen dioxide, particulate matter ≤ 2.5 µm, particulate matter ≤ 10 µm, and ozone), sleep (sleep duration) and socioeconomic status (combination of household income and parental education).

For the sensitivity analysis, different exposome scores were computed incorporating either a Mediterranean Diet Score or Baltic Sea Diet Score instead of the Finnish Children Healthy Eating Index to measure diet quality.

FCHEI: Finnish Children Healthy Eating Index, BSDS: Baltic Sea Diet Score, MDS: Mediterranean Diet Score, LC-MS: Liquid chromatography-mass spectrometry

Supplementary Figure S2. Comparison of the significant associations of exposome models using different diet scores in the formulation of the exposome score with serum metabolites measured by NMR

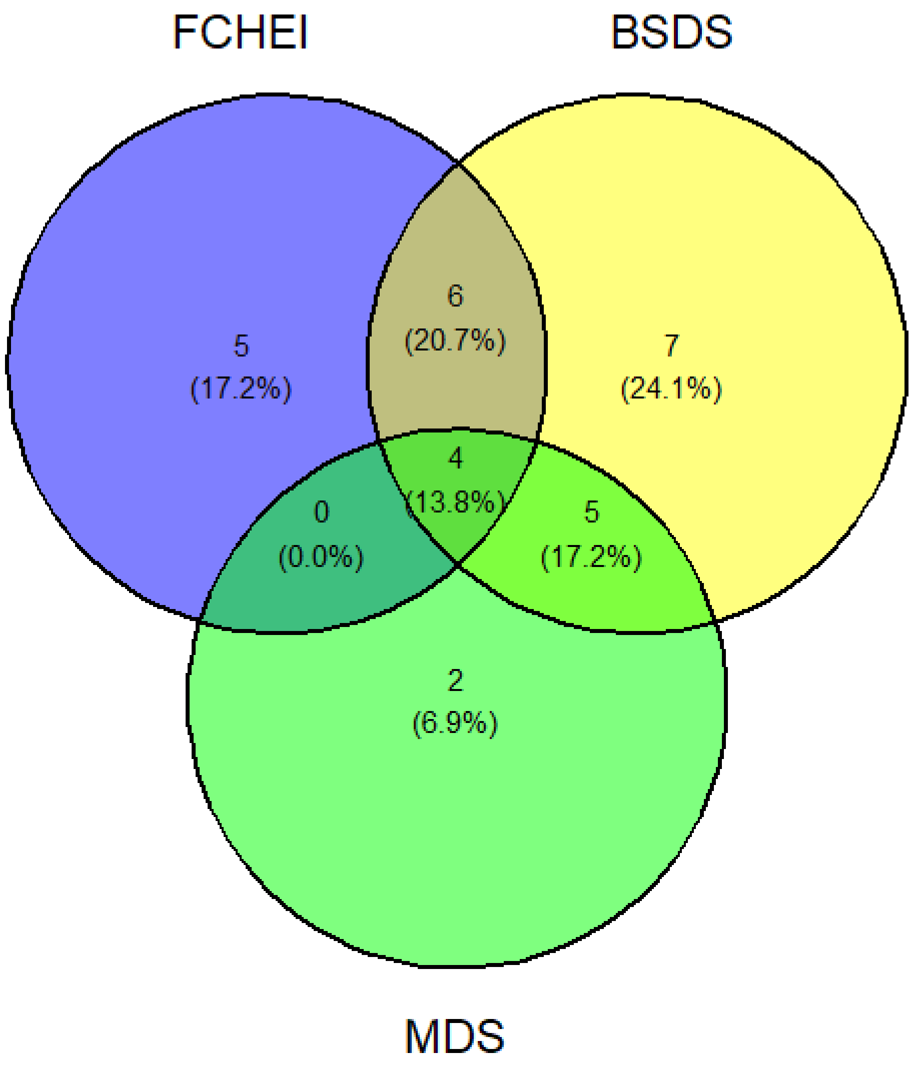

The exposome score (ranging between 5 and 20) was computed by summing up quartile scores for diet quality (measured by Finnish children healthy eating index), activity (combination of total physical activity and sedentary time), pollution exposure (combination of nitrogen oxide, nitrogen dioxide, particulate matter ≤ 2.5 µm, particulate matter ≤ 10 µm, and ozone), sleep (sleep duration) and socioeconomic status (combination of household income and parental education).
For the sensitivity analysis, different exposome scores were computed incorporating either a Mediterranean Diet Score or Baltic Sea Diet Score instead of the Finnish Children Healthy Eating Index to measure diet quality.
FCHEI: Finnish Children Healthy Eating Index, BSDS: Baltic Sea Diet Score, MDS: Mediterranean Diet Score, NMR: Nuclear Magnetic Resonance

Supplementary Figure S3. Intersections of the significant associations of the exposome score and leave-one-out models with serum metabolites measured by LC-MS

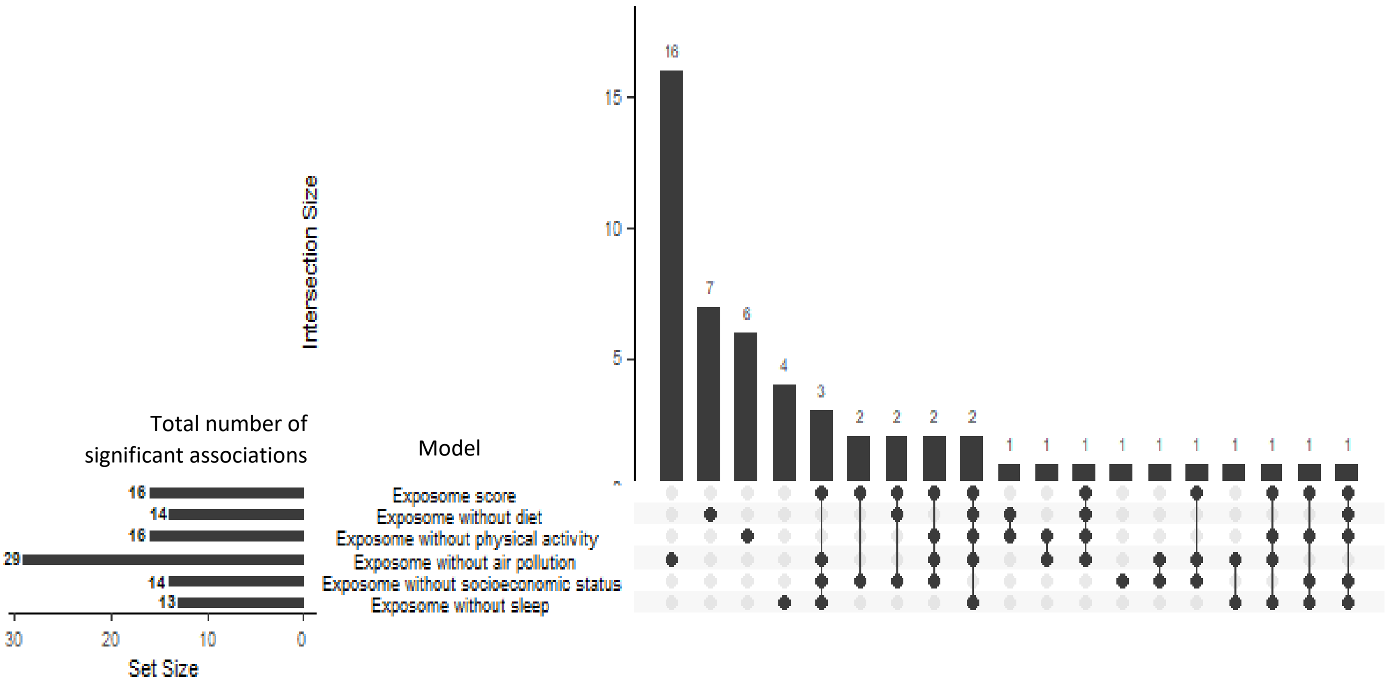

The exposome score (ranging between 5 and 20) was computed by summing up quartile scores for diet quality (measured by Finnish children healthy eating index), activity (combination of total physical activity and sedentary time), pollution exposure (combination of nitrogen oxide, nitrogen dioxide, particulate matter ≤ 2.5 μm, particulate matter ≤ 10 μm, and ozone), sleep (sleep duration) and socioeconomic status (combination of household income and parental education).

For the sensitivity analysis, five "leave-out" models were computed, removing one exposure category from the exposome score for each model. For example, the "Exposome without diet" model was calculated by computing the exposome score without a score for diet quality.

The overlap of significant associations of each model with serum metabolites measured are presented to the right of each model.

Supplementary Figure S4. Intersections of the significant associations of the exposome score and leave-one-out models with serum metabolites measured by NMR

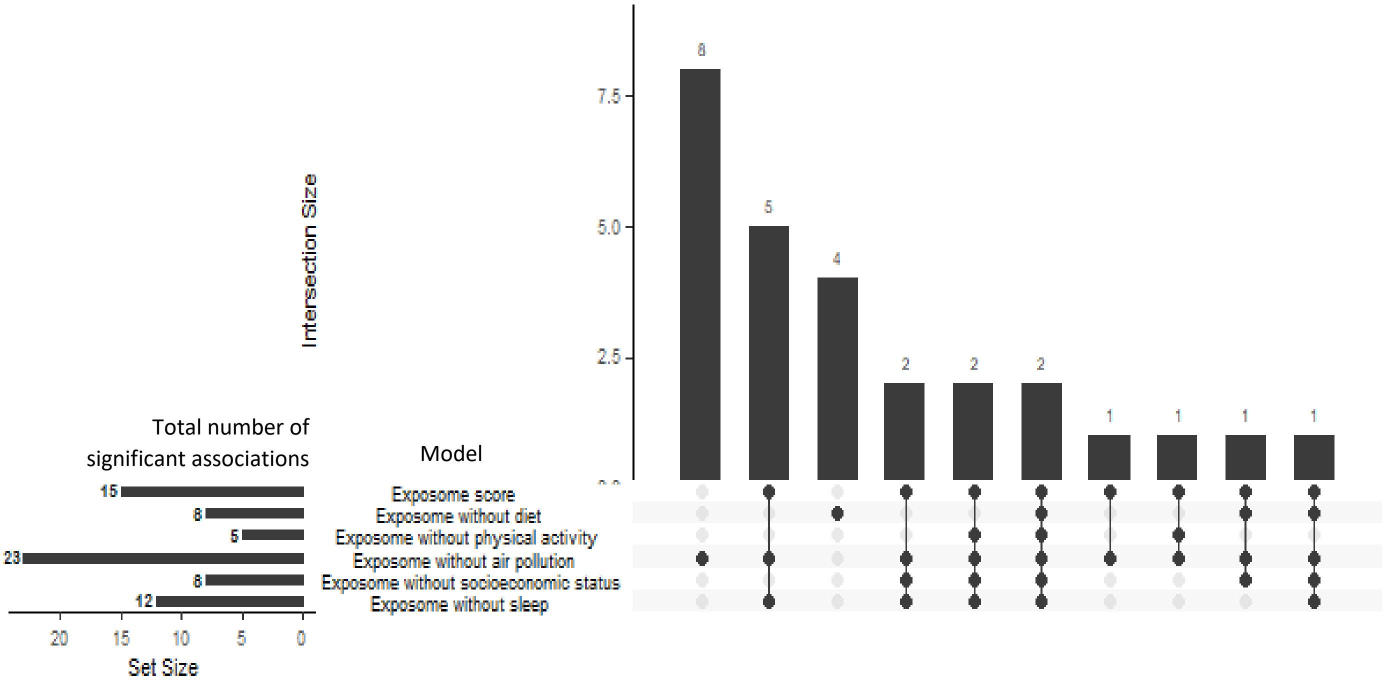

The exposome score (ranging between 5 and 20) was computed by summing up quartile scores for diet quality (measured by Finnish children healthy eating index), activity (combination of total physical activity and sedentary time), pollution exposure (combination of nitrogen oxide, nitrogen dioxide, particulate matter ≤ 2.5 µm, particulate matter ≤ 10 µm, and ozone), sleep (sleep duration) and socioeconomic status (combination of household income and parental education).

For the sensitivity analysis, five "leave-out" models were computed, removing one exposure category from the exposome score for each model. For example, the "Exposome without diet" model was calculated by computing the exposome score without a score for diet quality.

The overlap of significant associations of each model with serum metabolites measured are presented to the right of each model.
