# Supplementary material for: Longitudinal associations of an exposome score with serum metabolites from childhood to adolescence
Source: Commun Biol. 2024 Jul 22;7:890. doi: 10.1038/s42003-024-06146-0 (PMC11263428; doi:10.1038/s42003-024-06146-0)
Supplement: Supplementary file 3 — Description of Additional Supplementary Files [file 42003_2024_6146_MOESM3_ESM.pdf]

Description of Additional Supplementary Files

File name: Supplementary Data 1
Description: Associations of the exposome score with serum metabolites measured by LC-MS.

File name: Supplementary Data 2
Description: Associations of the exposome score with serum metabolites measured by NMR.

File name: Supplementary Data 3
Description: Comparison of the associations of the exposome score and its individual scores with serum metabolites measured by LC-MS.

File name: Supplementary Data 4
Description: Comparison of the associations of the exposome score and its individual scores with serum metabolites measured by NMR.

File name: Supplementary Data 5
Description: BMI-SDS as a modifier for the associations of the exposome score with serum metabolites measured by LC-MS.

File name: Supplementary Data 6
Description: BMI-SDS as a modifier for the associations of the exposome score with serum metabolites measured by NMR.

File name: Supplementary Data 7
Description: Leave-one-out analysis; comparison of the significant associations of the exposome score and leave-out models with serum metabolites measured by LC-MS.

File name: Supplementary Data 8
Description: Leave-one-out analysis; comparison of the significant associations of the exposome score and leave-out models with serum metabolites measured by NMR.
