# Supplementary material for: Longitudinal associations of an exposome score with serum metabolites from childhood to adolescence
Source: Commun Biol. 2024 Jul 22;7:890. doi: 10.1038/s42003-024-06146-0 (PMC11263428; doi:10.1038/s42003-024-06146-0)
Supplement: Supplementary file 5 — Reporting Summary [file 42003_2024_6146_MOESM5_ESM.pdf]

nature portfolio

Corresponding author(s): Darren Healy

Last updated by author(s): Jan 25, 2024

# Reporting Summary

Nature Portfolio wishes to improve the reproducibility of the work that we publish. This form provides structure for consistency and transparency in reporting. For further information on Nature Portfolio policies, see our Editorial Policies and the Editorial Policy Checklist.

## Statistics

For all statistical analyses, confirm that the following items are present in the figure legend, table legend, main text, or Methods section.

| n/a | Confirmed | |
|---|---|---|
| ☐ | ☒ | The exact sample size (*n*) for each experimental group/condition, given as a discrete number and unit of measurement |
| ☐ | ☒ | A statement on whether measurements were taken from distinct samples or whether the same sample was measured repeatedly |
| ☐ | ☒ | The statistical test(s) used AND whether they are one- or two-sided<br>*Only common tests should be described solely by name; describe more complex techniques in the Methods section.* |
| ☐ | ☒ | A description of all covariates tested |
| ☐ | ☒ | A description of any assumptions or corrections, such as tests of normality and adjustment for multiple comparisons |
| ☐ | ☒ | A full description of the statistical parameters including central tendency (e.g. means) or other basic estimates (e.g. regression coefficient) AND variation (e.g. standard deviation) or associated estimates of uncertainty (e.g. confidence intervals) |
| ☐ | ☒ | For null hypothesis testing, the test statistic (e.g. *F*, *t*, *r*) with confidence intervals, effect sizes, degrees of freedom and *P* value noted<br>*Give P values as exact values whenever suitable.* |
| ☒ | ☐ | For Bayesian analysis, information on the choice of priors and Markov chain Monte Carlo settings |
| ☒ | ☐ | For hierarchical and complex designs, identification of the appropriate level for tests and full reporting of outcomes |
| ☒ | ☐ | Estimates of effect sizes (e.g. Cohen's *d*, Pearson's *r*), indicating how they were calculated |

*Our web collection on statistics for biologists contains articles on many of the points above.*

## Software and code

Policy information about availability of computer code

| | |
|---|---|
| Data collection | No software was used |
| Data analysis | R, 2021, and RStudio, an Integrated Development Environment for R, version 2022.7.1.554. Mixed models were carried out with "lmer". The "performance" package was used to assess model diagnostics. The "interactions" package was used to plot effect modification. |

For manuscripts utilizing custom algorithms or software that are central to the research but not yet described in published literature, software must be made available to editors and reviewers. We strongly encourage code deposition in a community repository (e.g. GitHub). See the Nature Portfolio guidelines for submitting code & software for further information.

## Data

Policy information about availability of data

All manuscripts must include a data availability statement. This statement should provide the following information, where applicable:

- Accession codes, unique identifiers, or web links for publicly available datasets
- A description of any restrictions on data availability
- For clinical datasets or third party data, please ensure that the statement adheres to our policy

Information about the PANIC study and the variables used in the present paper are described at www.panicstudy.fi/en/etusivu. The data are not publicly available due to research ethical reasons and because the owner of the data is the University of Eastern Finland and not the research group. However, the corresponding author can provide further information on the PANIC study and the PANIC data on a reasonable request.

## Research involving human participants, their data, or biological material

Policy information about studies with human participants or human data. See also policy information about sex, gender (identity/presentation), and sexual orientation and race, ethnicity and racism.

| | |
|---|---|
| Reporting on sex and gender | Gender was not considered or reported on in this secondary analysis. Sex of the children was recorded at baseline with this assignment maintained at further timepoints.<br><br>At baseline, there were 243 (48.0%) females and 261 (52.0%) males. At 2-year follow-up, there was 214 (49.0%) females and 223 (51.0%) males. At 8-year follow-up, there was 126 (45.0%) females and 151 (55.0%) males.<br><br>Sex was adjusted for in the mixed models when investigation associations of the exposome score with serum metabolites. |
| Reporting on race, ethnicity, or other socially relevant groupings | Two socioeconomic status variables were measured in this study - parental education and household income. These were self-reported via questionnaire.<br><br>Parental education was split into 3 categories: vocational school or less, vocational high school, or university. Household income was split into 3 categories: <€30,000, €30,000 - €60,000, >€60,000.<br><br>These variables were incorporated into the "exposome score" as a category of interest, so were not adjusted for in the models. |
| Population characteristics | At baseline, the mean age was 7.63 ± 0.39. 97.6% of participants were assessed as Tanner Stage 1 (pubertal status). By the 8-year follow-up, 92% were either Tanner Stage 4 or 5. The mean standardised BMI was -0.17, -0.13 and -0.05 at timepoints 0-, 2- and 8-years, respectively. |
| Recruitment | We invited 736 children aged 6–9 years who started the first grade in 16 primary schools of the city of Kuopio in 2007–2009 to participate in the study. Altogether, 512 (70%) children (248 girls, 264 boys) accepted the invitation and participated in the baseline examinations between October 2007 and December 2009. The participants did not differ in sex, age, height-SD score (SDS) or BMI-SDS from all children who started the first grade in the city of Kuopio in 2007–2009. We excluded six children from the study at baseline either owing to their physical disabilities that could hamper participation in the intervention or withdrawal of the families because they had no time or motivation to attend the study. We also excluded data from two children whose parents or caregivers later withdrew their permission to use these data in the study. The final study sample thus included 504 children at baseline. |
| Ethics oversight | The Research Ethics Committee of the Hospital District of Northern Savo approved the study protocol in 2006 (Statement 69/2006). The PANIC study is registered at ClinicalTrials.gov with the identifier NCT01803776. |

Note that full information on the approval of the study protocol must also be provided in the manuscript.

# Field-specific reporting

Please select the one below that is the best fit for your research. If you are not sure, read the appropriate sections before making your selection.

☒ Life sciences ☐ Behavioural & social sciences ☐ Ecological, evolutionary & environmental sciences

For a reference copy of the document with all sections, see nature.com/documents/nr-reporting-summary-flat.pdf

# Life sciences study design

All studies must disclose on these points even when the disclosure is negative.

| | |
|---|---|
| Sample size | Sample size calculations were based the effects of a dietary intervention on fasting serum insulin and HOMA-IR among children in the Special Turku Coronary Risk Factor Intervention Project (STRIP; Kaitosaari et al., 2006). Because of a larger number of children in our study than in the STRIP study, we approximated a slightly smaller difference for the change in fasting serum insulin and HOMA-IR of 0.3 SD between the intervention group (60% of children) and the control group (40% of children) with a power of 80% and a two-tailed p-value for the difference between the groups of 0.05, allowing for a 20% loss to follow-up or missing data. However, these calculations did not allow for non-independence within schools, and therefore the power could be lower than 80%. According to our calculations, we resulted in a sample size of at least 275 children in the intervention group and at least 183 children in the control group at baseline. |
| Data exclusions | We excluded six children from the study at baseline either owing to their physical disabilities that could hamper participation in the intervention or withdrawal of the families because they had no time or motivation to attend the study. We also excluded data from two children whose parents or caregivers later withdrew their permission to use these data in the study. |
| Replication | Replication of findings has not yet taken place in another study/population. |
| Randomization | This was a non-randomised controlled trial. We allocated the children from nine schools to a combined physical activity and dietary intervention group (306 children, 60%) and the children from seven schools to a control group (198 children, 40%) to avoid contamination in the control group by any local or national health promotion programmes that could have been initiated in the study region during the follow-up period. We also proportionally matched the intervention and control group according to the location of the schools (urban vs rural) to |

| | |
|---|---|
| | minimise sociodemographic differences between the groups. We included more children in the intervention group than in the control group because of a larger number of dropouts expected in the intervention group and to retain a sufficient statistical power for comparison between the groups. |
| Blinding | The children, their parents or caregivers, or people carrying out the examination visits or doing the measurements were not blinded to the group assignment. Given that the intervention was lifestyle counselling, and the control group had no intervention, it was not possible to blind.<br><br>During data analysis for this manuscript, both groups (intervention and control) were pooled together. |

# Reporting for specific materials, systems and methods

We require information from authors about some types of materials, experimental systems and methods used in many studies. Here, indicate whether each material, system or method listed is relevant to your study. If you are not sure if a list item applies to your research, read the appropriate section before selecting a response.

## Materials & experimental systems

| n/a | Involved in the study | |
|---|---|---|
| ☒ | ☐ | Antibodies |
| ☒ | ☐ | Eukaryotic cell lines |
| ☒ | ☐ | Palaeontology and archaeology |
| ☒ | ☐ | Animals and other organisms |
| ☐ | ☒ | Clinical data |
| ☒ | ☐ | Dual use research of concern |
| ☒ | ☐ | Plants |

## Methods

| n/a | Involved in the study | |
|---|---|---|
| ☒ | ☐ | ChIP-seq |
| ☒ | ☐ | Flow cytometry |
| ☒ | ☐ | MRI-based neuroimaging |

# Clinical data

Policy information about clinical studies

All manuscripts should comply with the ICMJE guidelines for publication of clinical research and a completed CONSORT checklist must be included with all submissions.

| | |
|---|---|
| Clinical trial registration | clinicaltrials.gov NCT01803776 |
| Study protocol | www.panicstudy.fi |
| Data collection | Data collection was conducted in the city of Kuopio, Finland, with baseline examinations between October 2007 and December 2009. 2-year and 8-year follow-up examinations took place between 2009 and 2011, and 2015 and 2017, respectively. |
| Outcomes | For this secondary analysis, the primary outcome was associations of the developed exposome score with serum metabolites measured by LC-MS and NMR. Secondary outcomes included a comparison of the significant findings associated with the exposome score with associations from individual categories (diet, activity, air pollution, sleep and socioeconomic stress). Furthermore, the associations of the exposome score with serum metabolites was further investigated to determine if adiposity mediated any of the relationships, or if it acted as an effect modifier. |
